# Effect of Food Restriction on Adipose Tissue in Spontaneously Diabetic Torii Fatty Rats

**DOI:** 10.1155/2009/715057

**Published:** 2009-08-19

**Authors:** Hisayo Morinaga, Takeshi Ohta, Kenichi Matsui, Tomohiko Sasase, Sumiaki Fukuda, Makoto Ito, Masatoshi Ueda, Yukihito Ishii, Katsuhiro Miyajima, Mutsuyoshi Matsushita

**Affiliations:** ^1^Japan Tobacco Inc., Central Pharmaceutical Research Institute, 1-1, Murasaki-cho, Takatsuki, Osaka 569-1125, Japan; ^2^Japan Tobacco Inc., Toxicology Research Laboratories, Central Pharmaceutical Research Institute, 23 Naganuki, Hadano, Kanagawa 257-0024, Japan

## Abstract

Spontaneously Diabetic Torii-*fa/fa* (SDT fatty) rat is a new model of obese type 2 diabetes. SDT fatty rat exhibits obesity associated with hyperphagia. In this study, SDT fatty rats were subjected to pair-feeding with SDT-+/+ (SDT) rats from 6 to 22 weeks of age. The ratio of visceral fat weight to subcutaneous fat weight (V/S) decreased at 12 weeks of age in the pair-feeding rats. The intraperitoneal fat weight such as epididymal and retroperitoneal fat weight decreased, whereas mesenteric fat weight had no change. Cell size of the epididymal fat in the pair-feeding rats tended to decrease. Glucose oxidation level in epididymal fat in the pair-feeding rats at 12 weeks of age was recovered to a similar level with that in SDT rats. These results indicated that SDT fatty rat is a useful model to evaluate the functional or the morphological features in adipose tissue and develop a novel drug for antiobesity.

## 1. Introduction

Spontaneously Diabetic Torii (SDT) fatty rat is a new model of obese type 2 diabetes, established by introducing the *fa* allele of the Zucker fatty rat into the SDT rat genome [1]. SDT rat is a useful model of nonobese type 2 diabetes that spontaneously develops hyperglycemia and glucose intolerance resulting from decreased insulin secretion due to *β*-cell degeneration [2, 3]. It is worthy of notice that SDT rat shows ocular complications such as venous dilation and meandering vascular networks [4]. SDT fatty rat exhibited hyperphagia and obesity immediately after weaning, and the diabetes and its complications are found at a younger age in SDT fatty rats than in SDT rats [5].

Obesity plays a key role in the pathophysiology of several metabolic diseases and is a risk factor for diabetes mellitus or dyslipidemia. It has been reported that the distribution of fat between subcutaneous and visceral sites affects its metabolic impact. Specifically, increased abdominal adiposity has been identified as a risk factor for diabetes mellitus [6–9]. Aging in rats is associated with increased fasting and postprandial plasma insulin levels, suggesting an insulin-resistant state. The insulin resistance has a correlation with increase of fat weight, especially visceral fat [10, 11]. Food intake in SDT fatty rats at 6 weeks of age showed about twofold increase as compared with that in SDT rats, and body mass index and fat pad weight in SDT fatty rats at 14 weeks of age increased [1]. In SDT fatty rat, which shows hyperphagia and obesity, it is essential that the relationships of the changes between food intake and fat tissue are examined in detail. The present study was conducted to investigate how food restriction in SDT fatty rats affects fat weight, fat mass, or metabolic function of adipose tissue.

## 2. Materials and Methods

### 2.1. Animals and Diet

This experiment was conducted in compliance with the Guidelines for Animal Experimentation of Japan Tobacco biological/pharmacological research laboratories. Male SDT-*fa*/*fa* (SDT fatty) rats and age-matched SDT-+/+ (SDT) rats from our colonies were used. SDT fatty rats at 6 weeks of age were divided into two groups. One group was allowed feed (CRF-1, Charles River Japan, Yokohama, Japan) *ad libitum*, and the other group was pair-fed the same amount of food consumed by age-matched SDT rats from 6 to 22 weeks of age. Food consumption in the pair-fed SDT fatty rats was about 50%–60% of that in the *ad libitum*-fed SDT fatty rats during the experimental period. In brief, three groups of rat at 6 weeks of age were prepared: (a) SDT fatty rats fed *ad libitum* (Fatty Group), (b) SDT fatty rats pair-fed against SDT rats (Fatty-PF Group), and (c) SDT rats fed *ad libitum* (SDT Group). A satellite group of each of the three groups was established to evaluate additional effects to 12 weeks of age. The rats were housed individually in suspended bracket cages in a climate-controlled room with a temperature of 23 ± 3°C, humidity of 55 ± 15%, and a 12 hours lighting cycle and had free access to water.

### 2.2. Biological Parameters

Body weights and blood chemical parameters, such as glucose, triglyceride (TG), insulin, leptin, and adiponectin levels, were measured at 6, 12, and 22 weeks of age.

Blood samples were collected from the tail vein of rats. Serum glucose and TG levels were measured using commercial kits (Roche Diagnostics, Basel, Switzerland) and an automatic analyzer (Hitachi 7180; Hitachi, Tokyo, Japan). Serum insulin or leptin levels were measured with a rat insulin- or leptin enzyme-linked immunosorbent assay (ELISA) kit (Morinaga Institute of Biological Science, Yokohama, Japan). Serum adiponectin was measured with a mouse/rat adiponectin ELISA kit (Otsuka pharmaceutical Inc., Tokyo, Japan).

### 2.3. Fat Tissue Weight

Visceral and subcutaneous fat weights in each rat were determined at 6, 12, and 22 weeks of age by computed tomography (CT) analysis. The fat weights were measured by a laboratory X-ray CT device (LATheta, ALOKA Co., LTD., Osaka, Japan). Rats were anesthetized with an intraperitoneal injection of 50 mg/kg pentobarbital (Tokyo chemical industry, Tokyo, Japan), and about 20 CT photographs per rat were taken at 5 mm intervals between diaphragm and lumbar vertebrae. The ratio of Visceral and Subcutaneous (V/S) were calculated. 

Necropsy was performed in satellite groups at 12 weeks of age, and weights of intraperitoneal fat, such as epididymal, retroperitoneal, and mesenteric fat, were determined. Rats were sacrificed by exsanguination under light ether anesthesia, and intraperitoneal fats were collected and weighed. Epididymal fats obtained in this necropsy were used in evaluation of fat cell size and in glucose oxidation and mRNA expression experiments. Since a sufficient fat amount is easily collected, epididymal fats were used in those experiments.

### 2.4. Fat Cell Size

Cell size of epididymal fat was determined at 12 weeks of age. Cell number of fixed view area was counted for 3 different areas per rat. Cells with incomplete shape on the view frame were not counted. Cells size was estimated as the fixed area divided by the cell number.

### 2.5. Glucose Oxidation

Glucose oxidation levels on epididymal fat were determined at 12 weeks of age. Adipose tissue samples were incubated in Hanks' balanced salt solution (pH 7.4, GIBCO, Grand Island, NY, USA) containing 4% BSA (bovine serum albumin, Sigma Chemical, St. Louis, MO, USA), 20.72 kBq/mL [U]-^14^C-glucose (NEN, Boston, MA, USA), and 0–100 mU/mL insulin (from bovine pancreas, Sigma) for 120 minutes at 37°C. Synthesized ^14^CO_2_ was trapped with Scintilamine-OH (Dojindo, Tokyo, Japan) on a filter paper (Whatman, Maidstone, UK) and counted using a Liquid Scintillation Analyzer Model 2500 TR (Packard, Meriden, CT, USA). Total lipid synthesis from glucose was estimated by determining the amount of ^14^C incorporated into total lipids. Total lipids were extracted from the tissues with n-hexane after the addition of Dole's solution (2-propanol : n-heptane : 1 N H_2_SO_4_ = 40 : 10 : 1). Radioactivity in the hexane fractions was measured using a liquid scintillation counter.

### 2.6. mRNA Quantification with Real-Time Quantitative PCR

Total RNA was extracted from the epididymal fat of the satellite group rats at 12 weeks of age. RNA was transcribed into cDNA using M-MLV reverse transcriptase and random primers (Invitrogen, Carlsbad, CA, USA). The reaction mixture was incubated for 10 minutes at 25°C, 1 hour at 37°C, and 5 minutes at 95°C. Real time PCR quantification was performed in a 50 *μ*L reaction mixture with an automated sequence detector combined with ABI Prism 7700 Sequence Detection System software (Applied Biosystems, Foster City, CA, USA). The reaction mixture contained 50 ng of synthesized cDNA, 3.5 mM MgCl_2_, 0.3 *μ*M primers, 0.1 *μ*M probes, and 1.25 units of Ampli Taq Gold. Cycle parameters were 10 minutes at 95°C, followed by 40 cycles of 15 seconds at 95°C and 60 seconds at 60°C. The following primers and FAM-conjugated probes were designed using Primer Express software (Applied Biosystems): glucose transporter 4 (GLUT4) (forward, CTACATCCGAACCTGGAG; reverse, AGTGCATCAGACACATCAGCCC; probe, TGCCCGAAA GAGTCT AAA GCG), tumor necrosis factor *α* (TNF*α*) (forward, CCAGGTTCTCTTCAAGGGACAA; reverse, CTCCTGGTATGAAATGGCAAATC; probe, CCCGACTATGTGTGCTCCTCACCCACA), lipoprotein lipase (LPL) (forward, TGTCTAACTGCCACTTCAACCACA; reverse, CATACATTCCTGTCACCGTCCA; probe, CAGCAA AACCTTTGTGGTGATCCATGG), Acetyl CoA carboxylase 1 (ACC-1) (forward, GCAGCTATGTTCAGAGAGTTCACC; reverse, CCA CCT CAC AGT TGA CTT GTT TTC; probe, CGGCGACTT ACGTTC CTA GTTGCA CAA AA) and 18 seconds rRNA (purchased from Applied Biosystems).

### 2.7. Statistical Analyses

Results are expressed as the mean ± standard deviation (SD). Statistical analysis of differences between mean values was performed using the F-test, followed by the Student's *t*-test or Aspin-Welch's *t*-test. Differences were defined as significant at *P* < .05.

## 3. Results

### 3.1. Biological Parameters

**Figure 1:**
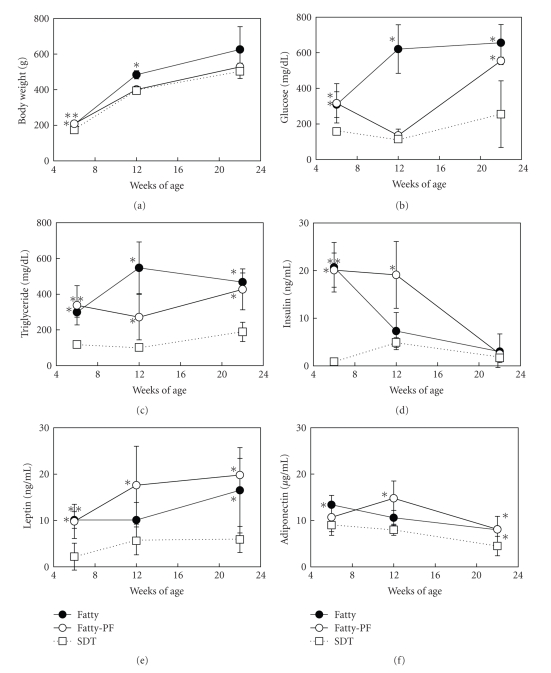
Changes in body weights (a) and serum parameters ((b) Glucose; (c) Triglyceride; (d) Insulin; (e) Leptin; (f) Adiponectin) in Fatty rats, Fatty-PF rats, and SDT rats. Body weight in Fatty-PF rats showed similar change to that in SDT rats. Hyperglycemia and hypertriglyceridemia in Fatty rats were temporally improved by food restriction, and the hyperinsulinemia was maintained to 12 weeks of age. Serum leptin and adiponectin levels were elevated in the Fatty-PF rats. Data represent means ± SD (*n* = 5-6). *P < .05, **P < .01: significantly different from SDT rat.

Body weight in the SDT fatty rats was already elevated as compared with that in the SDT rats at the starting point of this experiment, 6 weeks of age (mean value: SDT fatty rats, 207 g; SDT rats, 174 g). Body weight in the Fatty Group showed a significant increase at 12 weeks of age as compared with that in the SDT Group, whereas body weight in the Fatty-PF Group showed similar changes at 12 and 24 weeks of age to those in the SDT Group (Figure 1(a)).

Serum glucose level in the SDT fatty rats was already elevated as compared with that in the SDT rats at 6 weeks of age (mean value: SDT fatty rats, 313 mg/dL; SDT rats, 158 mg/dL). Serum glucose level in the Fatty Group was elevated to 600–700 mg/dL at 12 and 22 weeks of age. Serum glucose level in the Fatty-PF Group was suppressed to a similar level as in the SDT Group at 12 weeks of age, whereas the glucose level was elevated to about 550 mg/dL at 22 weeks of age (Figure 1(b)). Hypertriglyceridemia in the Fatty Group was sustained during the experimental period. Serum TG level in the Fatty-PF Group tended to be decreased at 12 weeks of age as compared with that in the Fatty Group (mean value: Fatty-PF Group, 271 mg/dL; Fatty Group, 548 mg/dL; see Figure 1(c)). Hyperinsulinemia in the Fatty Group was shown at 6 weeks of age, whereas, after 12 weeks of age, the insulin level decreased to a level similar to that in the SDT Group (Figure 1(d)). Hyperinsulinemia in the Fatty-PF Group was maintained until 12 weeks of age. Serum leptin level in the SDT fatty rats was elevated as compared with that in the SDT rats at 6 weeks of age (mean value: SDT fatty rats, 10.0 ng/mL; SDT rats, 2.2 ng/mL). The elevation in the Fatty-PF Group was sustained during the experimental period (Figure 1(e)). Serum leptin level in the Fatty Group also tended to increase. Serum adiponectin level in the Fatty-PF Group was significantly increased at 12 and 22 weeks of age (Figure 1(f)). The adiponectin level in the Fatty Group was significantly increased at 22 weeks of age, but the level at the starting point of this experiment tended to be increased as compared with that in the SDT Group.

Food restriction in SDT fatty rats induced a temporal improvement of hyperglycemia and an increase of serum leptin and adiponectin level.

### 3.2. Fat Tissue Weight

**Figure 2:**
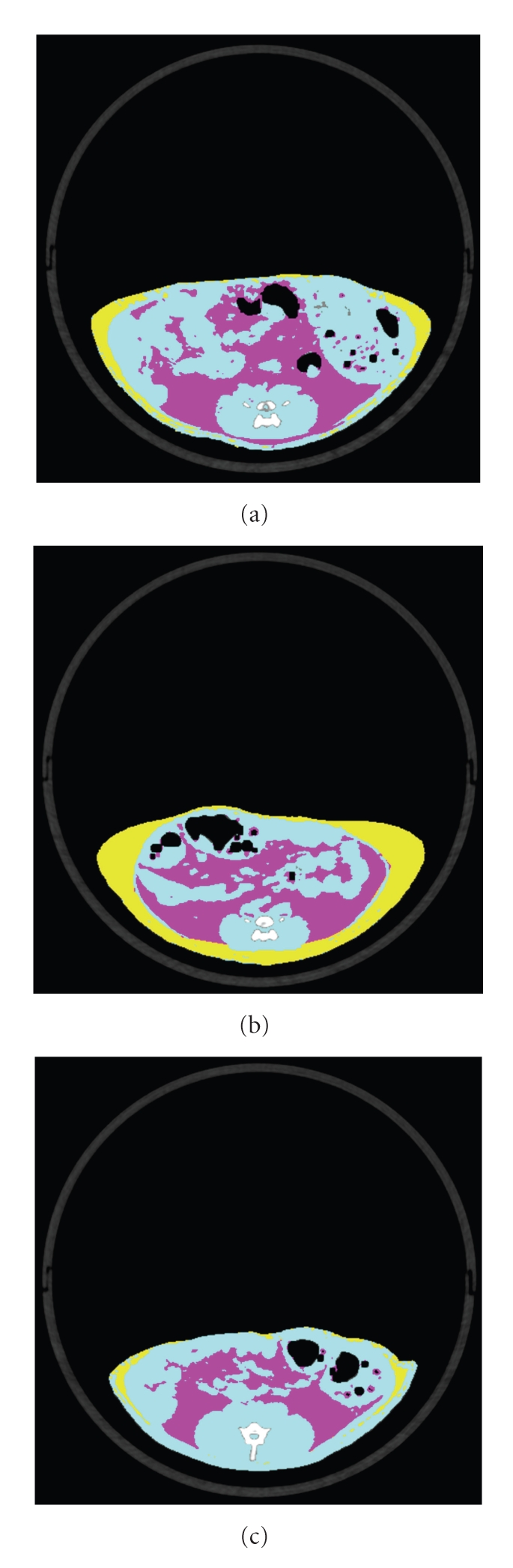
Computed tomography analysis at 12 weeks of age (representative tomogram). (a) Fatty rat; (b) fatty-PF rat; (c) SDT rat. Yellow: subcutaneous fat; red: visceral fat; blue: lean. Visceral fat area in fatty rat was decreased by food restriction.

At 6 weeks of age, total fat weight, which is the sum of visceral fat weight and subcutaneous fat weight, was about 5 times higher in the SDT fatty rats than in the SDT rats. The fat weight in each group was elevated with aging, from 6 to 22 weeks of age. The fat weights and V/S ratios at 6 weeks of age were comparable in the Fatty Group and the Fatty-PF Group. At 12 weeks of age, total fat weights in both groups were at similar levels (mean value: Fatty Group, 87.1 g; Fatty-PF Group, 87.8 g), but visceral fat weight and V/S ratio in the Fatty-PF Group were decreased as compared with those in the Fatty Group (mean value of visceral fat weight or V/S ratio: Fatty Group, 47.9 g or 1.29; Fatty-PF Group, 37.0 g or 0.75; see Table 1, Figure 2). Moreover, subcutaneous fat weight in the Fatty-PF Group was increased as compared with that in the Fatty Group. Similar changes were observed at 22 weeks of age, but the changes were not significant (Table 1).

In examination of intraperitoneal fat weight at 12 weeks of age, epididymal and retroperitoneal fat weights in the Fatty-PF Group were decreased at 12 weeks of age as compared with those in the Fatty Group, whereas the mesenteric fat weights between two groups were not changed (Table 2).

Food restriction in SDT fatty rats induced decreases of visceral fat weight and V/S ratio.

### 3.3. Fat Cell Size

**Figure 3:**
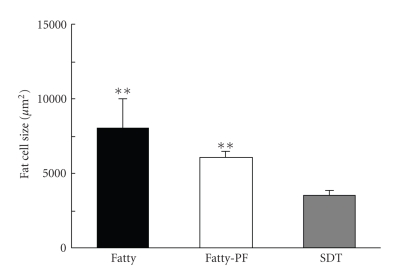
Fat cell size in epididymal fat at 12 weeks of age. Fat cell size in fatty rats was about 2.3 times higher than that in SDT rats. The fat cell size tended to decrease by about 25% by food restriction. Data represent means ± SD (*n* = 6). **P < .01: significantly different from SDT rat.

Fat cell size at 12 weeks of age in the Fatty Group was about 2.3 times higher than that in the SDT Group (mean ± SD: Fatty Group, 8059 ± 1967 *μ*m^2^; SDT Group, 3553 ± 341 *μ*m^2^). The fat cell size in the Fatty-PF Group (mean ± SD: 6080 ± 355 *μ*m^2^) tended to be decreased by about 25% as compared with that in the SDT Group (Figure 3).

Food restriction in SDT fatty rats induced a miniaturization of the fat cell size.

### 3.4. Glucose Oxidation

**Figure 4:**
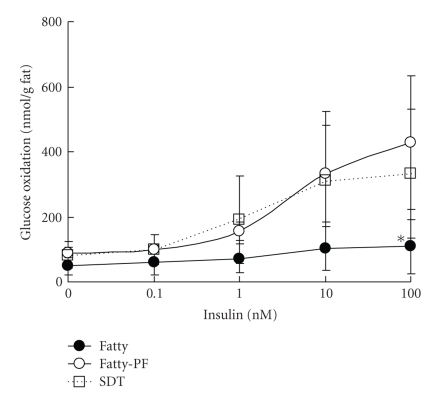
Glucose oxidation levels in epididymal fat at 12 weeks of age. The glucose oxidation levels were examined under basal- (without insulin) or insulin-stimulated (0.1–100 nM) conditions. Insulin-stimulated glucose oxidation in fatty rats was deteriorated as compared with that in SDT rats. The glucose oxidation level was recovered by food restriction. Data represent means ± SD (*n* = 6). *P < .05: significantly different from SDT rat.

Both basal glucose oxidation levels and insulin-stimulated glucose oxidation levels in adipose tissue of SDT fatty rats tended to be decreased as compared with those in SDT rats (Figure 4), and especially in 100 nM insulin, the decrease of glucose oxidation in the Fatty Group was significant. Glucose oxidation levels in the Fatty-PF Group were recovered to similar levels as in the SDT Group.

Food restriction in the SDT fatty rats induced improvement of the metabolic function of adipose tissue.

### 3.5. mRNA Expression in Adipose Tissue

**Figure 5:**
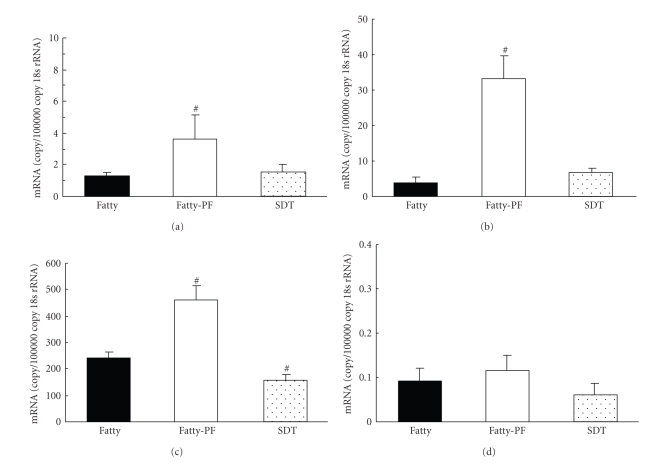
GLUT4 (a), ACC-1 (b), LPL (c) and TNF*α* (d) mRNA levels in epididymal fat at 12 weeks of age. All mRNA expressions were measured by a real-time quantitative PCR method. The mRNA levels were corrected with 18 seconds rRNA. Data represent means ± SD (*n* = 6). ^#^ *P* < .05: significantly different from SDT fatty rat.

GLUT4 and ACC-1 mRNA levels between the Fatty Group and the SDT Group were not changed, whereas those levels in the Fatty-PF Group were elevated as compared with that in the Fatty Group (Figures 5(a) and 5(b)). LPL mRNA level in the Fatty Group increased as compared with that in the SDT Group, and the levels in the Fatty-PF Group were, furthermore, elevated (Figure 5(c)). TNF*α* mRNA level in the Fatty Group tended to increase as compared with that in the SDT Group, whereas the levels between the Fatty Group and the Fatty-PF Group were not changed (Figure 5(d)).

## 4. Discussion

SDT rat, which is a model of nonobese type 2 diabetes, was established in 1997 by Shinohara et al. [2]. The rat spontaneously develops hyperglycemia and glucose intolerance resulting from decreased insulin secretion due to *β*-cell degeneration [12]. Interestingly, SDT rat shows ocular complications, such as venous dilation and meandering vascular networks, which are caused by hyperglycemia [4]. It is also reported that SDT rat is useful as a model of diabetic nephropathy [13]. More recently, Masuyama et al. [1] produced an SDT fatty rat by introducing the *fa* allele of the Zucker fatty rat into the SDT rat genome. SDT fatty rats manifest hyperphagia, obesity, hyperglycemia, and hyperlipidemia. The fatty rats develop diabetes from 5 weeks of age, a development time that is quite earlier than for SDT rats [5]. This early incidence or progression of diabetes is considered to be caused by hyperphagia, which is associated with a leptin signal abnormality induced by introduction of the *fa* allele. In this study, we investigated the effects of food restriction on bloodchemical parameters and adipose tissue in SDT fatty rats.

Under food restriction, body weights in the Fatty Group were regulated to similar levels as those in the SDT Group (Figure 1(a)). The rats in the Fatty-PF Group showed an improvement of hyperglycemia and a tendency of TG levels to be decreased at 12 weeks of age (Figures 1(b) and 1(c)). These improvements in glucose and TG levels were also reported under food-regulated conditions of Zucker diabetic fatty rats [14] and by food restriction in OLETF rats [15]. Moreover, food restriction in normal rats (Wistar rats) did not show a lowering effect on fasting plasma glucose level, whereas the fasting plasma insulin level decreased [16]. The improvement in the Fatty-PF Group was not continuously observed, and the elevation of glucose and TG levels was observed at 22 weeks of age (Figures 1(b)  and 1(c)). Since SDT fatty rats might have weakness in pancreatic function [1], it is considered that the insulin secretion is deteriorated, and the incidence of diabetes in the rats cannot be suppressed by food restriction alone. Serum insulin level in the Fatty-PF Group was decreased to a similar level in the SDT fatty Group at 22 weeks of age (Figure 1(d)). Also, elevation of TG levels at 22 weeks of age in the Fatty-PF Group is considered to be caused by the insulin deficiency. Both serum leptin level and serum adiponectin level in SDT fatty rats were increased by food restriction (Figures 1(e) and 1(f)). It is reported that plasma leptin level was decreased, and plasma adiponectin level was increased by food restriction in Wistar rats and OLETF rats [15, 16]. The change in adiponectin level was consistent with the result in our study, but the change in leptin level was inconsistent with our result. Since leptin resistance exists in SDT fatty rats, the serum leptin level may not be decreased by food restriction. Furthermore, the increase in serum leptin levels in food-restricted SDT fatty rat is considered to be caused by the increase of subcutaneous fat weight (Table 1). On the other hand, the increase of serum adiponectin levels in food-restricted SDT fatty rat is considered to be related to the decrease of visceral fat weight. In the investigation on the effects of food restriction on biochemical parameters in SDT fatty rats, a temporal improvement of hyperglycemia and an increase of adipokine level were observed. Especially, the improvement of hyperglycemia was partial, and the reason is considered to be related with the background factor in SDT rats.

In analysis of fat tissue weights in the pair-feeding SDT fatty rat, the visceral fat or the V/S ratio significantly decreased, whereas the subcutaneous fat tended to increase as compared with the SDT fatty rat (Table 1). It is reported that total fat weight, including visceral fat and subcutaneous fat, was decreased by food restriction in normal rats [16–18], OLETF rats [15], and Zucker fatty rats [19]. The change of visceral fat in our study was consistent with results in some reports, but the change of subcutaneous fat mass in our study was inconsistent with the some results. Those changes of fat tissue weight in this study were similar with changes after treatment with thiazolidinediones [20, 21]. After pioglitazone treatment, subcutaneous fat area increased, whereas visceral fat area and the ratio of visceral to subcutaneous fat decreased [20, 21]. In human obesity, the contribution of abdominal visceral and subcutaneous fat is widely noticed [22, 23]. There is a strong association between visceral adiposity and insulin resistance patterns of glucose homeostasis, including in type 2 diabetes mellitus [24, 25]. Furthermore, visceral fat is related to essential hypertension, dyslipidemia, and cardiovascular disease [26, 27]. Evaluation for the pattern of that fat distribution in SDT fatty rat is considered to be very important to elucidate pathophysiological features in human obesity.

According to CT analysis at 6, 12, and 22 weeks of age, we showed significant difference in the ratio of visceral fat to subcutaneous fat at 12 weeks of age (Table 1). So we investigated fat weight, fat cell size, glucose oxidation, and quantification of mRNA at this age. In examination of intraperitoneal fat weight, epididymal and retroperitoneal fat weights in the SDT fatty rats were decreased by food restriction for 6 weeks, whereas the mesenteric fat weight was not changed (Table 2). Epididymal and retroperitoneal fat weights in Wistar rats and OLETF rats had been decreased by food restriction [15, 16]. Furthermore, the mesenteric fat weight in OLETF rats was lowered by the food restriction for 12 weeks. A decrease of mesenteric fat weight may not be caused by food restriction for 6 weeks, which was a condition in our study.

Fat cell size in the SDT fatty rat tended to decrease by food restriction (Figure 3). It is reported that a decrease of energy intake, that is, a restriction of food intake, induces miniaturization of fat cells in Sprague-Dawley rats [28, 29]. In general, food restriction is considered to induce an increase in the number of small adipocytes in rats. Furthermore, it is reported that a miniaturization of fat cell size induces increase of plasma adiponectin levels [30]. It is possible that the increase of serum adiponectin levels in the pair-feeding rat (Figure 1(f)) was induced by the miniaturization of fat cell size.

Insulin stimulated-glucose oxidation in the pair-feeding SDT fatty rats was elevated as compared with that in the SDT fatty rats (Figure 4), and it is considered that the glucose metabolic function in the SDT fatty rat was promoted by food restriction. Glucose utilization index and glucose metabolic index of retroperitoneal and epididymal adipose tissues has been also increased in food-restricted Wistar rats [16, 17]. Since it is reported that insulin stimulated-glucose oxidation was elevated by a reinforcement of insulin sensitivity [31], the recovery of glucose oxidation in the food-restricted SDT fatty rats may be caused by the improvement of glicolipid abnormalities, such as the decrease of serum glucose level and TG level (Figures 1(b) and 1(c)). Also, increases of blood leptin level and adiponectin level are considered to induce good effects on the metabolic function of adipose tissue [16, 32–35]. Furthermore, GLUT4, LPL, and ACC mRNA expressions in the pair-feeding SDT fatty rats increased (Figure 5). It is reported that these mRNA expressions in adipose tissue is increased by insulin treatment [36, 37]. Since the serum insulin level in the pair-feeding SDT fatty rats at 12 weeks of age increased (Figure 1(d)), the elevation of these mRNA expressions is considered to be induced by the increase of the insulin levels. Furthermore, another reason for the elevation of these mRNA expressions is considered to be reinforcement of insulin sensitivity by food restriction (Figure 4). Decrease of TNF*α* mRNA expression and increase of GLUT4 mRNA expression were shown in treatment with thiazolidinediones [38, 39], but the TNF*α* mRNA expression did not change in this study (Figure 5(b)). Although this evaluation of adipose tissue was performed by using epididymal fats, the further examination by using other adipose tissue might be essential. In the investigation on the effects of food restriction on adipose tissue in SDT fatty rats, a decrease of visceral fat weight and a miniaturization of the fat cell size were observed. Moreover, the food restriction induced an improvement of the metabolic function on adipose tissue.

Food restriction in SDT fatty rats partially improved the glycolipid parameters and induced a decrease of the visceral fat weight. Furthermore, the food restriction affected the fat cell size and the metabolic function in the adipose tissue. In conclusion, SDT fatty rat is a useful model to evaluate the functional or the morphological features in adipose tissue and develop a novel drug for antiobesity.

## Figures and Tables

**Table 1:** Changes of fat tissue weights in Fatty Group, Fatty-PF Group, and SDT Group. Visceral and subcutaneous fat tissue weights were determined by computed tomography analysis. Data represent mean ± SD (*n* = 5-6).

	6 weeks of age	12 weeks of age	22 weeks of age
Fatty Group			
* *Visceral fat (g)	9.8 ± 1.0	47.9 ± 1.9	85.9 ± 15.8
* *Subcutaneous fat (g)	16.3 ± 1.7	39.2 ± 10.9	51.6 ± 37.3
* *Total fat (g)	26.1 ± 2.4	87.1 ± 11.0	137.5 ± 52.5
* *V/S ratio	0.60 ± 0.07	1.29 ± 0.31	2.04 ± 0.66
Fatty-PF Group			
* *Visceral fat (g)	10.5 ± 1.0	37.0 ± 1.4^*##*^	75.5 ± 2.5
* *Subcutaneous fat (g)	16.5 ± 1.9	50.8 ± 9.0	59.8 ± 8.8
* *Total fat (g)	27.1 ± 2.8	87.8 ± 9.2	135.2 ± 10.4
* *V/S ratio	0.64 ± 0.04	0.75 ± 0.15^*##*^	1.28 ± 0.19
SDT Group			
* *Visceral fat (g)	2.9 ± 0.8	17.8 ± 2.8	27.1 ± 10.6
* *Subcutaneous fat (g)	2.2 ± 0.6	9.1 ± 2.3	13.5 ± 6.2
* *Total fat (g)	5.1 ± 1.4	26.9 ± 5.0	40.6 ± 16.7
* *V/S ratio	1.31 ± 0.07	2.01 ± 0.22	2.30 ± 0.89

^*##*^ *P* < .01: significantly different from Fatty Group.

**Table 2:** Intraperitoneal fat tissue weights at 12 weeks of age. Data represent mean ± SD (*n* = 6).

	Epididymal fat (g)	Retroperitoneal fat (g)	Mesenteric fat (g)
Fatty Group	14.6 ± 0.7	16.4 ± 1.4	10.9 ± 0.6
Fatty-PF Group	12.0 ± 1.8^*##*^	12.8 ± 1.7^*##*^	10.7 ± 0.9
SDT Group	6.8 ± 0.7	8.7 ± 1.0	5.5 ± 0.6

^*##*^ *P* < .01: significantly different from Fatty Group.

## References

[1] Masuyama T, Katsuda Y, Shinohara M (2005). A novel model of obesity-related diabetes: introgression of the *Lepr*
^*fa*^ allele of the Zucker fatty rat into nonobese Spontaneously Diabetic Torii (SDT) rats. *Experimental Animals*.

[2] Shinohara M, Masuyama T, Shoda T (2000). A new spontaneously diabetic non-obese torii rat strain with severe ocular complications. *Experimental Diabetes Research*.

[3] Masuyama T, Fuse M, Yokoi N (2003). Genetic analysis for diabetes in a new rat model of nonobese type 2 diabetes, Spontaneously Diabetic Torii rat. *Biochemical and Biophysical Research Communications*.

[4] Sasase T, Ohta T, Ogawa N (2006). Preventive effects of glycaemic control on ocular complications of Spontaneously Diabetic Torii rat. *Diabetes, Obesity and Metabolism*.

[5] Matsui K, Ohta T, Oda T (2008). Diabetes-associated complications in Spontaneously Diabetic Torii fatty rats. *Experimental Animals*.

[6] Peiris AN, Struve MF, Mueller RA, Lee MB, Kissebah AH (1988). Glucose metabolism in obesity: influence of body fat distribution. *Journal of Clinical Endocrinology and Metabolism*.

[7] Bjorntorp P (1991). Metabolic implications of body fat distribution. *Diabetes Care*.

[8] Kissebah AH (1991). Insulin resistance in visceral obesity. *International Journal of Obesity*.

[9] Carey DG, Jenkins AB, Campbell LV, Freund J, Chisholm DJ (1996). Abdominal fat and insulin resistance in normal and overweight women: direct measurements reveal a strong relationship in subjects at both low and high risk of NIDDM. *Diabetes*.

[10] Barzilai N, Banerjee S, Hawkins M, Chen W, Rossetti L (1998). Caloric restriction reverses hepatic insulin resistance in aging rats by decreasing visceral fat. *The Journal of Clinical Investigation*.

[11] Gabriely I, Ma XH, Yang XM, Rossetti L, Barzilai N (2002). Leptin resistance during aging is independent of fat mass. *Diabetes*.

[12] Masuyama T, Komeda K, Hara A (2004). Chronological characterization of diabetes development in male Spontaneously Diabetic Torii rats. *Biochemical and Biophysical Research Communications*.

[13] Ohta T, Matsui K, Miyajima K (2007). Effect of insulin therapy or renal changes in spontaneously diabetic Torii rats. *Experimental Animals*.

[14] Colombo M, Kruhoeffer M, Gregersen S (2006). Energy restriction prevents the development of type 2 diabetes in Zucker diabetic fatty rats: coordinated patterns of gene expression for energy metabolism in insulin-sensitive tissues and pancreatic islets determined by oligonucleotide microarray analysis. *Metabolism*.

[15] Kimura M, Shinozaki T, Tateishi N (2006). Adiponectin is regulated differently by chronic exercise than by weight-matched food restriction in hyperphagic and obese OLETF rats. *Life Sciences*.

[16] Escrivá F, Gavete ML, Fermín Y (2007). Effect of age and moderate food restriction on insulin sensitivity in Wistar rats: role of adiposity. *Journal of Endocrinology*.

[17] Sugden MC, Grimshaw RM, Lall H, Holness MJ (1994). Regional variations in metabolic responses of white adipose tissue to food restriction. *American Journal of Physiology*.

[18] Hynes GR, Heshka J, Chadee K, Jones PJ (2003). Effects of dietary fat type and energy restriction on adipose tissue fatty acid composition and leptin production in rats. *Journal of Lipid Research*.

[19] Azain MJ, Hausman DB, Kasser TR, Martin RJ (1995). Effect of somatotropin and feed restriction on body composition and adipose metabolism in obese Zucker rats. *American Journal of Physiology*.

[20] Kelly IE, Han TS, Walsh K, Lean MEJ (1999). Effects of a thiazolidinedione compound on body fat and fat distribution of patients with type 2 diabetes. *Diabetes Care*.

[21] Miyazaki Y, Mahankali A, Matsuda M (2002). Effect of pioglitazone on abdominal fat distribution and insulin sensitivity in type 2 diabetic patients. *Journal of Clinical Endocrinology and Metabolism*.

[22] Kelley DE, Thaete FL, Troost F, Huwe T, Goodpaster BH (2000). Subdivisions of subcutaneous abdominal adipose tissue and insulin resistance. *American Journal of Physiology*.

[23] Ross R, Aru J, Freeman J, Hudson R, Janssen I (2002). Abdominal adiposity and insulin resistance in obese men. *American Journal of Physiology*.

[24] Despres J-P, Nadeau A, Tremblay A (1989). Role of deep abdominal fat in the association between regional adipose tissue distributioin and glucose tolerance in obese women. *Diabetes*.

[25] Colberg SR, Simoneau J-A, Thaete FL, Kelley DE (1995). Skeletal muscle utilization of free fatty acids in women with visceral obesity. *Journal of Clinical Investigation*.

[26] Bjorntorp P “Portal” adipose tissue as a generator of risk factors for cardiovascular disease and diabetes.

[27] Despres J-P (1993). Abdominal obesity as important component of insulin-resistance syndrome. *Nutrition*.

[28] Pedersen O, Kahn CR, Flier JS, Kahn BB (1991). High fat feeding causes insulin resistance and a marked decrease in the expression of glucose transporters (glut 4) in fat cells of rats. *Endocrinology*.

[29] Cha MC, Jones PJH (1998). Dietary fat type and energy restriction interactively influence plasma leptin concentration in rats. *Journal of Lipid Research*.

[30] Okuno A, Tamemoto H, Tobe K (1998). Troglitazone increases the number of small adipocytes without the change of white adipose tissue mass in obese Zucker rats. *Journal of Clinical Investigation*.

[31] Shibata T, Matsui K, Yonemori F, Wakitani K (1998). JTT-501, a novel oral antidiabetic agent, improves insulin resistance in genetic and non-genetic insulin-resistant models. *British Journal of Pharmacology*.

[32] Scarpace PJ, Nicolson M, Matheny M (1998). UCP2, UCP3 and leptin gene expression: modulation by food restriction and leptin. *Journal of Endocrinology*.

[33] Fernández-Galaz C, Fernández-Agulló T, Pérez C (2002). Long-term food restriction prevents ageing-associated central leptin resistance in wistar rats. *Diabetologia*.

[34] Zhou H, Song X, Briggs M (2005). Adiponectin represses gluconeogenesis independent of insulin in hepatocytes. *Biochemical and Biophysical Research Communications*.

[35] Yang G, Li L, Tang Y, Boden G (2006). Short-term pioglitazone treatment prevents free fatty acid-induced hepatic insulin resistance in normal rats: possible role of the resistin and adiponectin. *Biochemical and Biophysical Research Communications*.

[36] Sivitz WI, DeSautel SL, Lee EC, Pessin JE (1992). Time-dependent regulation of rat adipose tissue glucose transporter (GLUT4) mRNA and protein by insulin in streptozocin-diabetic and normal rats. *Metabolism*.

[37] Becker DJ, Ongemba LN, Brichard V, Henquin J-C, Brichard SM (1995). Diet- and diabetes-induced changes of ob gene expression in rat adipose tissue. *FEBS Letters*.

[38] Ahuja HS, Liu S, Crombie DL (2001). Differential effects of rexinoids and thiazolidinediones on metabolic gene expression in diabetic rodents. *Molecular Pharmacology*.

[39] Furuta M, Yano Y, Gabazza EC (2002). Troglitazone improves GLUT4 expression in adipose tissue in an animal model of obese type 2 diabetes mellitus. *Diabetes Research and Clinical Practice*.

